# Efficacy and well-being in rural north India: The role of social identification with a large-scale community identity

**DOI:** 10.1002/ejsp.2060

**Published:** 2014-08-25

**Authors:** Sammyh S Khan, Nick Hopkins, Shruti Tewari, Narayanan Srinivasan, Stephen David Reicher, Gozde Ozakinci

**Affiliations:** 1 Medical School, University of ExeterExeter, UK; 2 School of Psychology, University of DundeeDundee, UK; 3 Centre of Behavioural and Cognitive Sciences, University of AllahabadAllahabad, India; 4 School of Psychology & Neuroscience, University of St. AndrewsSt Andrews, UK; 5 School of Medicine, University of St AndrewsSt Andrews, UK

## Abstract

Identifying with a group can contribute to a sense of well-being. The mechanisms involved are diverse: social identification with a group can impact individuals' beliefs about issues such as their connections with others, the availability of social support, the meaningfulness of existence, and the continuity of their identity. Yet, there seems to be a common theme to these mechanisms: identification with a group encourages the belief that one can cope with the stressors one faces (which is associated with better well-being). Our research investigated the relationship between identification, beliefs about coping, and well-being in a survey (N = 792) administered in rural North India. Using structural equation modelling, we found that social identification as a Hindu had positive and indirect associations with three measures of well-being through the belief that one can cope with everyday stressors. We also found residual associations between participants' social identification as a Hindu and two measures of well-being in which higher identification was associated with poorer well-being. We discuss these findings and their implication for understanding the relationship between social identification (especially with large-scale group memberships) and well-being. We also discuss the application of social psychological theory developed in the urban West to rural north India. © 2014 The Authors. *European Journal of Social Psychology* published by John Wiley & Sons, Ltd.

The concept of social identity offers insights into how social processes impact positively on health and well-being (Haslam, 2014; Haslam, Jetten, Postmes, & Haslam, 2009; Jetten, Haslam, & Haslam, 2012). Although the mechanisms involved in the relationship between social identification and well-being are diverse, it seems they entail a common theme: identifying with social groups is associated with the belief that one can deal with stressful challenges. In what follows, we examine how and why social identity processes may be relevant to well-being and whether identifying with a large-scale social group membership is associated with such benefits. We then investigate this issue empirically with questionnaire data from rural north India. We ask if identifying as a Hindu is associated with well-being *via* its associations with believing that one can deal with the challenges of everyday life (what we term ‘stress-related self-efficacy’). Specifically, we test a model in which social identification as a Hindu has an indirect and positive association with well-being *via* greater stress-related self-efficacy. As we will see in the succeeding text, the fact that we investigated identification with a large-scale group, and that we did so in India, gives these questions additional interest.

## Social Identification and Well-being

It is well known that the more social relationships and the more networks one has, the better one's health and well-being (Cohen, 2004; Helliwell & Putnam, 2004; Kawachi, Subramanian, & Kim, 2008; Smith & Christakis, 2008). The social identity perspective to group behavior enriches this literature in various ways. It argues we can think of ourselves in different ways: sometimes in terms of our personal identities and sometimes in terms of social group memberships (Turner, Hogg, Oakes, Reicher, & Wetherell, 1987). These latter can be small-scale face-to-face groups (e.g., a work team) but also large-scale (e.g., a nation: Anderson, 1983).

The consequences of such social identifications are several and have various implications for well-being. Most obviously, thinking of oneself and others as sharing a group membership implies that these others are one's fellows, and this increases the degree to which people help each other (Levine, Prosser, Evans, & Reicher, 2005; Wakefield et al., 2011). Moreover, on the basis of one's common social identification, one expects to be recognized as a fellow group member (Hopkins & Greenwood, 2013) and to receive the benefits (including social support from one's fellows) that come with such membership (Haslam, Reicher, & Levine, 2011). In turn, the more one identifies with a group and the more one expects social support from other group members, so the better one's well-being in the face of intense stress (Haslam, O'Brien, Jetten, Vormedal, & Penna, 2005; Wegge, Van Dick, Fisher, Wecking, & Moltzen, 2006). Research also shows that it is the level of one's subjective identification with the group rather than the actual level of contact with other group members that predicts well-being (Sani, Herrera, Wakefield, Boroch, & Gulyas, 2012). Moreover, research shows that manipulating people's awareness of the number of their group memberships can impact upon people's resilience in the face of immediate stressors: with more group memberships psychologically salient, participants exhibit greater resilience (Jones & Jetten, 2011). Again, the implication is that social identifications facilitate coping.

Given such potential benefits, it is appropriate to consider the types of social identification associated with improved well-being. Not all will: most obviously, some group identifications are associated with unhealthy practices and norms that de-value health as a goal (Tarrant & Butler, 2011). Moreover, group identifications may have contradictory implications. For example, even if associated with unhealthy practices, a social identification may still bring benefits in the social support it allows one to access. Furthermore, with regards to the potential benefits, it is important to consider the issue of group size and scale. Thus far, most research has tended to focus on small groups whose immediate circumstances are extremely challenging (e.g. groups of bomb-disposal experts; see Haslam et al., 2005), and it is not immediately obvious that larger-scale identifications would have similar implications nor that they would facilitate coping with more everyday stressors. This is the focus for our own research.

## Social Groups: Scope and Scale

Large-scale identifications (e.g., national or religious) differ from face-to-face groups (e.g., work teams) in various ways (e.g., entitativity: Lickel et al., 2000) and can satisfy different identity motives (Easterbrook & Vignoles, 2012). The mechanisms by which large-scale social identifications could be associated with well-being are diverse. On the negative side, some cultural norms may encourage unhealthy practices (e.g., diet: Guendelman, Cheryan, & Monin, 2011) or discourage certain medical interventions (e.g., the use of Allopathic medicine: Thomas, 1992). On the positive side, some cultural traditions may provide a sense of trans-historical continuity (Sani et al., 2007) in a way that work teams may not. As work on national identifications has shown, such continuity can provide a psychologically comforting sense of meaning (Sani, Bowe, & Herrera, 2008; Sani, Herrera, & Bowe, 2009). Moreover, there is evidence that identifying with larger-scale groups can facilitate resilience in the face of immediate stressors: in the aforementioned work by Jones and Jetten (2011) showing that one's awareness of the number of one's group memberships affects resilience, some of the memberships involved were large-scale (e.g. gender).

The benefits of identifying with a large-scale group membership are also suggested in studies that show those who are religious tend to fare better in terms of health than their non-religious counterparts (George, Ellison, & Larson, 2002). In part, this is because certain religious traditions discourage practices that bring health risks (e.g., alcohol consumption). It can also be because religious beliefs and cultural practices encourage ways of explaining life experiences, which limit the impact of negative events (Ano & Vasconcelles, 2005; James & Wells, 2003; Seeman, Dubin, & Seeman, 2003). However, it is also because being religious implies a social identification with a group (Ysseldyk, Matheson, & Anisman, 2010). This group identification can bind one to (apparently ‘eternal’) cultural traditions (which may reduce existential anxiety; Kinnvall, 2004). It can also bind one to fellow believers in supportive social networks—for example, church congregations (George, Larson, Koenig, & McCullough, 2000; George et al., 2002; Lim & Putnam, 2010). Moreover, such is the scale of the identification (and the range of people to which it makes a connection at least potentially possible), that it not only connects one to an existing network, but also provides the basis for linking one to new networks if old ones should be disrupted. This is apparent in work by Ysseldyk, Haslam, and Haslam (2013) who found that among older adults undergoing significant life-course transitions (e.g., moving into a residential home), a religious identification allowed individuals to more easily join and establish new face-to-face groups (thereby renewing their social capital).

In the current research, we explored further this potential for large-scale social identifications to be associated with better well-being. On the basis of previous research, it appears that social identifications may provide individuals with a sense of continuity, purpose and meaning, social connection with others, and access to social support, and we asked if identifying with a large-scale social group may therefore predict people's beliefs about the degree to which they can cope with stressful situations (stress-related self-efficacy). That is, we asked if large-scale social identifications have a wider association with well-being beyond the immediate and known demands of particular occupations (Haslam et al., 2005) or particular physical circumstances (Jones & Jetten, 2011) and whether this is bound up with people's beliefs about their general ability to deal with the various unpredictable demands thrown up in everyday life.

Our research is distinctive in another way, too: it was conducted in rural north India. Thus far, most work on the association between social identification and well-being has been conducted in urban settings in the industrialized West, and the degree to which such research is relevant to other sites (and the everyday stressors that characterize them) cannot be assumed. Accordingly, our research site has particular merits: it allows analysis of the relevance of identifying with a group for coping with the everyday stressors that are integral to rural life in a developing country. To find relationships between social identification, stress-related self-efficacy, and well-being in such a setting would be good evidence for the wider applicability of recent theorizing on the social bases of well-being (Jetten et al., 2012).

## Community Identity in Rural North India

Social identities in rural north India are complex. There are local occupation-based caste identities with their own histories and local social significance (Gupta, 2005). There are also larger-scale community identities such as ‘Hindu’. Although factors associated with social coordination in complex societies help explain the cultural evolution of such identities (Shariff, 2011), historical research shows that this identity takes its modern form and significance because British colonial administrators sought to delineate and manage the constituent elements of Indian society (Freitag, 1980; Thapar, 1999).

In contemporary India, this Hindu identity permeates the fabric of everyday life. It has spiritual and religious elements, yet also constitutes a cultural identity (Doniger, 2010) such that all aspects of life, from who lives where in a village to whether and how one relates to others, are informed by this identity. Thus, for a Hindu to self-define strongly in terms of this collective identity is not simply to subscribe to a set of spiritual beliefs but to define the nature of one's connections to others in one's everyday transactions.

Investigating the association of such a social identification with well-being is complicated. There are questions concerning psychology's approach to culture and cultural differences (Misra & Gergen, 2002). These can be particularly important when studying health and well-being. Stress-related symptoms can be somatised differently in different cultures (Hwang, Myers, Abe-Kim, & Ting, 2008; Kirmayer & Young, 1998), and this highlights the need for culturally-appropriate measures of well-being. Other issues concern the nature and significance of the social relationships found in different cultures. Researchers often differentiate between individualistic and collectivist cultures and an important aspect of this contrast concerns cultural differences in the weightings people give to information provided by knowledge of an individual's group memberships and family connections. For example, Owe et al. (2013) show such information to be more important in collectivist cultures. Moreover, in different cultures there may be different ways in which social relationships inform one's identity (Becker et al., 2012), and there is evidence that in some collectivist cultures (e.g., Japan), group memberships tend to be depicted in terms of networks of interrelated individual members (Yuki, 2003; Yuki, Maddux, Brewer, & Takemura, 2005).

However, despite such potential cross-cultural complexities, there are reasons to believe the social identity concept can be useful in non-Western contexts. Although there may be different bases for group entitativity in non-Western contexts, Yuki et al. (2005) observe that this does not undermine the significance of group memberships for cognition and behavior. Yet, it remains important to recognize the cultural meanings of the relevant identities (Hopkins & Reicher, 2005). For example, although it is common to assume that a shared social identification implies that group members are seen as interchangeable exemplars of a horizontal community (the paradigmatic example being European national identities: Anderson, 1983), this should not be presumed to be a universal characteristic of social identities (Yuki, 2003; Yuki et al., 2005). In India, a common Hindu identification can exist alongside hierarchical caste categorizations, and although these latter remain bases for differentiation, this does not necessarily subvert a superordinate (highly entitative) Hindu identification. Indeed, caste identities are routinely represented as complementary and as allowing the Hindu ‘body’ to function (Prayag Magh Mela Research Group, 2007).

Another issue relating to the application of well-being research conducted in the industrialized West to rural north India concerns the social significance of religion. Most research on religiosity and well-being has been conducted with Christian denominations in the US. This has prompted calls for a wider range of religious identifications to be studied (George et al., 2002). This requires cultural sensitivity. For example, it would be easy to assume that as public Christian prayer typically involves a set service at which a congregation gathers (therefore, providing opportunities for sustained social interaction), then prayer at a Hindu temple has similar implications. However, this is misleading (the concept of set services is less applicable), and attendance at Hindu temples cannot be taken as a measure of network participation. It is also important to note that in religious countries (and India is one of the most religious countries: see PEW, 2002), religious believers are socially valued, and such social value can impact upon the degree to which people receive various psychological benefits (e.g., social self-esteem, psychological adjustment; see Gebauer, Sedikides, & Neberich, 2012). In other words, some issues that are important in US Christian communities (e.g., congregational prayer) may be less important in Hindu practice, and other issues of lesser significance in more secular cultures (e.g., the status accorded to the religious) may be more important in India.

## The Current Research

Our research involved individuals who were, in formal terms, Hindus, and explored if and how their subjective identification with this large-scale social group was associated with their well-being (whether reported in psychological terms, e.g., as ‘anxiety’, or in somatised terms, e.g., as ‘body-aches’: Pereira et al., 2007). In particular, we addressed the question of whether this identification was indirectly associated with better well-being *via* its association with stress-related self-efficacy (the belief that one can deal with the challenges of everyday life). As far as we are aware, there is no research in India exploring this relationship (see Sharma & Sharma, 2010).

Given the complex associations of a Hindu identification in India (e.g., with religious practice and with social status), our analyses also took into account two other factors, which could be relevant to well-being. The first concerned participants' level of engagement in religious practices. The second was participants' sense of their social standing in the community (relevant because the religious are accorded greater social value in religious societies: Gebauer et al., 2012).

In addition to addressing these two key alternative predictors of stress-related self-efficacy, our analyses of the relationship between social identification as a Hindu, stress-related self-efficacy, and well-being also controlled for a number of background socio-demographic variables. Specifically, we controlled for the effects of age, gender, caste, and marital status, which Indian research shows are relevant to health and well-being (e.g., Baru, Acharya, Acharya, Kumar, & Nagaraj, 2010; Borooah, 2010; Jensen, 2005; Mohindra, Haddad, & Narayana, 2006; Mukherjee, Haddad, & Narayana, 2011). We also controlled for educational-level (which in India can have unique effects on well-being beyond poverty; Rajan, Kennedy, & King, 2013).

## METHOD

### Sample

Participants (*N* = 792) were recruited in the rural area within a radius of 100–120 km from Allahabad (Uttar Pradesh, Northern India). We approached known local contacts and recruited others through a process of snowballing. All participants self-categorized as Hindu. The average age was 64.1 years (*SD* = 10.75). Three hundred seventy-three (47.1%) were female and 419 (52.9%) were male. Seven hundred nineteen (90.8%) belonged to the *general caste* category and 73 (9.2%) to the *other backward caste* category (OBC). These broad caste categories are used by the Indian Government to differentiate between those who are relatively privileged (general caste) and those who are less so (OBC). Other caste categories below OBC exist but were not represented in our sample. Six hundred twenty-seven (79.2%) were married and 165 (20.8%) were widowed. 313 were illiterate (39.5%), 380 (48%) had primary-to-intermediate education, and 99 (12.5%) were university educated.

This is not a representative sample of the north Indian population. Rather, it is an older and higher-caste sample, and this is because we targeted villagers considering an age-related pilgrimage (Tewari, Khan, Hopkins, Srinivasan, & Reicher, 2012). However, a relatively homogenous sample is entirely suitable for our attempt to investigate a process model of identification and well-being.

### Design and Procedure

Our data derive from an orally-administered (Hindi) questionnaire. The scales were developed through extensive piloting and were translated and back-translated by two independent groups. Any differences in the translations were resolved by improving the items. The final items were piloted again with illiterate and literate Hindi-speakers.

The questionnaires were administered by a team of 10 Hindi-speaking field investigators in participants' homes. Completion of each questionnaire took approximately 30 minutes. So as to make the concept of a five-point scale meaningful, we showed participants drawings of five glasses containing increasing levels of water (ranging from empty to full; see Tewari et al., 2012) and explained how participants could communicate the level to which they agreed with the statements they would hear (through pointing to the relevant glass).

When approaching potential participants, the researchers identified themselves as coming from the University of Allahabad and as being interested in villagers' lives and experiences. The researchers gave an overview of the questionnaire's contents and addressed any questions that potential participants raised. After this, consent was sought (“Do we have your consent to participate in this survey study?”). Given literacy issues, the explanation of the research, the request for consent, and the giving of consent was oral (this was approved by the Ethics Committees of the University of Dundee and the University of Allahabad). No incentives were offered for participation.

### Measures

Our questionnaire measured participants': (i) level of social identification as a Hindu; (ii) perceptions of efficacy in dealing with everyday stressors; (iii) well-being; (iv) engagement in religious practices; and (v) perceptions of their social standing in their community. Except where stated otherwise, all items were answered on five-point scales using the drawings of glasses of water explained earlier. The scale names and their items are as follows:

#### Social Identification as Hindu

Three items asked: ‘To what extent does being Hindu matter to you?’; ‘To what extent is being Hindu a key part of your life?’, and ‘To what extent is being a Hindu central to your sense of who you are?’ Responses were anchored: 1 = ‘Not at all’; 5 = ‘Completely’ (which conceptually translates into English as ‘A lot’).

#### Stress-Related Self-Efficacy

Five items based on Schwarzer and Jerusalem's Generalized Self-Efficacy Scale (1995) but modified for use in India. Using the stem ‘Over the last week, to what extent have you been feeling you…’, the items included ‘can manage all the demands on you?’, ‘have the capabilities to do the things that matter to you?’, ‘can manage your life well?’, ‘are in control of your life?’, and ‘have the skill/abilities to live your life as you want?’ (anchored: 1 = ‘Not at all’; 5 = ‘Completely’).

#### Well-being

This was measured using various scales. The first (labeled *self-assessed health*) comprised three items from the internationally-used core module of the Centers for Disease Control and Prevention Health Related Quality of Life Measure (CDC HRQOL-14; 2000). Using the stem ‘Over the last week, how would you describe your…’, the items included ‘physical health’; ‘state of mind’; and ‘energy levels?’ (anchored: 1 = ‘Very Poor’; 5 = ‘Very Good’). We also included items adapted from scales developed for use in the Indian Subcontinent, which take account of the somatisation of stress-related symptoms (Ruback, Begum, Tariq, Kamal, & Pandey, 2002; Ruback, Pandey, & Begum, 1997). Using the stem ‘Over the last week, to what extent have you felt…’, three questions concerned psychological symptoms (‘anxious without any reason?’, ‘restless without any reason?’, and ‘irritable without any reason?’; anchored: 1 = ‘Not at all’; 5 = ‘Completely’). Three others concerned physical symptoms (using the stem ‘Over the last week, to what extent have you suffered from…’ these items referred to ‘body-aches and pains?’, ‘breathlessness?’, and ‘headaches?’; anchored: 1 = ‘Not at all’; 5 = ‘Completely’). These six items were formed into two scales (labeled psychological symptoms of ill-health and physical symptoms of ill-health, respectively).

#### Religious Practices

Six items addressed participants' level of engagement in religious practices. Three items concerned Religious Practices at Home. Using the stem ‘In the last week, how often have you…’ the questions were ‘performed morning pujas (prayers)?’, ‘performed evening pujas?’, and ‘chanted religious texts in your home?’ Using the same stem, three concerned Religious Practices in Temples and asked how often they had ‘gone to temples?’, ‘offered fruits/sweets/flowers in the temple?’, and ‘read or chanted religious texts in the temple?’ For these items, participants reported how many days over the last week they had engaged in each practice (with 0 indicating none and every subsequent unit representing one day per week to a maximum of 7).

#### Perceived Standing in the Group

Five items asked about the extent to which participants thought that other people in their neighborhood or village treated them as members of standing in the village community. Using the stem ‘Currently, to what extent do others like your neighbours and other villagers…’ the items included: ‘accord you high status?’, ‘like you?’, ‘take you seriously?’, ‘admire you?’, ‘respect you?’ (anchored: 1 = ‘Not at all’; 5 = ‘Completely’).

We assessed if these items formed constructs in the intended manner using Confirmatory Factor Analysis in AMOS 17.0 (Arbuckle, 2008). We specified a factor structure with eight latent variables that were allowed to co-vary freely. Symptoms of ill-health were specified as two discrete factors: psychological symptoms of ill-health and physical symptoms of ill-health. Likewise, religious practices at home and religious practices in temples were specified as two separate factors. The chi-square goodness of fit statistic for the specified factor structure was significant *χ*^2^ (322) = 914.37, p < .001). However, with a sample of this size, this does not necessarily mean the model should be rejected (e.g., Bentler, 1990; Jöreskog & Sörbom, 1996). Additionally, we used the comparative fit index (CFI), the root mean squared error of approximation (RMSEA) and the standardized root mean squared residual (SRMR) to evaluate model fit. It has been proposed that values of >0.95 for the CFI (Hu & Bentler, 1999), <0.05 for the RMSEA (Browne & Cudeck, 1993), and < 0.05 for the SRMR (Byrne, 2010) indicate a good fit between a specified model and observed data. Given these recommendations, our specified factor structure had a good fit (CFI = 0.95, RMSEA = 0.05 (90% CI: 0.045–0.052), SRMR = 0.03). The measurement weights for the respective latent variables specified in the CFA are displayed in Table 1.^1^

**Table 1:** Measurement weights from confirmatory factor analysis

Latent variables and items	β
Social identification as a Hindu	
To what extent:	
-Does being Hindu matter to you?	0.80***
-Is being Hindu a key part of your life?	0.92***
-Is being a Hindu central to your sense of who you are?	0.83***
Religious practices at home	
In the last week:	
-How often have you performed morning pujas?	0.63***
-How often have you performed evening pujas?	0.61***
-How often have you chanted religious texts in your home?	0.62***
Religious Practices in Temples	
In the last week:	
-How often have you gone to temples?	0.94***
-How often have you offered fruits/sweets/flowers in the temple?	0.94***
-How often have you read or chanted religious texts in the temple?	0.66***
Perceived standing in the group	
Currently, to what extent do others like your neighbors and other villagers:	
-Accord you high status?	0.88***
-Like you?	0.89***
-Take you seriously?	0.88***
-Admire you?	0.83***
-Respect you?	0.84***
Stress-related self-efficacy	
Over the last week, to what extent have you been feeling you:	
-Can manage all the demands on you?	0.67***
-Have the capabilities to do the things that matter to you?	0.80***
-Can manage your life well?	0.87***
-Are in control of your life?	0.77***
-Have the skill/abilities to live your life as you want?	0.79***
Self-assessed health	
Over the last week, how would you describe your:	
-Physical health?	0.72***
-State of mind?	0.75***
-Energy levels?	0.72***
Psychological symptoms of ill-health	
In the last week, how often have you felt :	
-Anxious without any reason?	0.79***
-Restless without any reason?	0.86***
-Irritable without any reason?	0.73***
Physical symptoms of ill-health	
In the last week, how often have you suffered from:	
-Body-aches and pains?	0.68***
-Breathlessness?	0.46***
-Headaches?	0.63***

*Note*:

*p < .05. **p < .01 ***p < .001

Background information about participants' scores on these scales (in which the relevant item scores were averaged) appear in Table 2. This depicts the scale means, standard deviations, *Cronbach* alphas, and inter-scale correlations. The reliabilities for all measures apart from two were ≥0.78. The reliabilities for the religious practices at home were rather lower (0.62) than for those in the Temple (0.88)—probably because home-based prayer is rather more variable (e.g., not every home-based prayer would entail chanting). The overall reliability of the Physical Symptoms of Ill-Health scale was also low (0.61). However, the fact we have three measures of well-being means that this is less of an issue that would otherwise be. All measures were positively scored except symptoms of ill-health (where higher scores indicate more symptoms of ill-health). The inter-scale correlations (also available in Table 2) show that higher Social Identification as a Hindu was associated with better self-assessed health (but had no association with the psychological or the physical symptoms of ill-health). Table 2 also shows that greater stress-related self-efficacy was associated with higher involvement in religious practices (both at the home and at the temple), higher perceived standing in the group, and higher social identification as a Hindu.

**Table 2:** Means, standard deviations, Cronbach's Alphas and correlations

			2.	3.	4.	5.	6.	7.	8.	9.	10.	11.	12.	13.
	*M* (*SD*)	α	r	r	r	r	r	r	r	r	r	r	r	r
1. Social identification as a Hindu	4.73(0.52)	0.89	.14***	.08*	.19***	.27***	.14***	−.02	−.04	−.03	.08*	.14***	−.03	.11**
2. Religious practices at home	4.92(2.28)	0.62		36***	.08*	.20***	.16***	−.14***	−.17***	.08*	.26***	.10**	−.03	.33***
3. Religious practices in temples	2.76(2.86)	0.88			.09*	.14***	.09**	−.04	−.05	.03	.04	.02	−.02	.07
4. Perceived standing in the group	4.05(0.85)	0.95				.38***	.16***	.13***	.10**	−.02	−.09**	.14***	−.04	.03
5. Stress-related self-efficacy	3.79(0.93)	0.89					.47***	−.40***	−.38***	−.13***	.15**	.15***	.03	.27***
6. Self-assessed health	3.31(0.93)	0.78						−.48***	−.50***	−.18***	.27***	.03	.11**	.28***
7. Psychological symptoms of ill-health	2.15(1.13)	0.84							.58***	.04	−.29***	−.02	−.07*	−.29***
8. Physical symptoms of ill-health	2.02(0.89)	0.61								.10**	−.33***	−.06	−.03	−.35***
9. Age											.21***	.02	−.20***	−.04
10. Gender												−.03	.12**	.62***
11. Caste													−.02	.14***
12. Marital status														.13***
13. Education														

*Note*:

* p < .05.

** p < .01

*** p < .001;

Female, low-caste, widowed and illiterate participants were coded zero

## RESULTS

Our analyses entailed two steps. The first investigated if stress-related self-efficacy was associated with participants' social identification as a Hindu. The second involved testing a model in which Social Identification as a Hindu was associated with well-being *via* stress-related self-efficacy. In both analyses, we controlled for effects associated with the socio-demographic categories recorded (age, gender, caste, marital status, and education). Moreover, given the complex meaning of a Hindu identification, we also investigated the degree to which a Hindu social identification had associations with well-being that were distinct from those associated with participants' level of religious practices (at temples and at home) and perceived standing in the group. Both analyses employed Structural Equation Modelling (SEM; in AMOS 17.0, Arbuckle, 2008). Given the equivalence of our measures across socio-demographic categories (see footnote 1), these analyses used pooled data.

### Factors Associated with Stress-Related Self-Efficacy

Our first analysis specified a model in which participants' level of social identification as a Hindu, level of religious practices (at temples and at home), and perceived standing in the group were entered as predictors of stress-related self-efficacy. The socio-demographic categories were entered as control variables (with female, lower caste, widowed, and illiterate participants coded zero). Accordingly, stress-related self-efficacy was the only endogenous variable with the others being exogenous (and allowed to co-vary freely with one another).

The chi-square goodness of fit statistic for the model was significant (*χ*^2^ (212) = 771.57, p < .001). The remaining goodness of fit indices were satisfactory (CFI = 0.95; RMSEA = 0.06 (90% CI: 0.053–0.062); SRMR = 0.04). Table 3 summarizes the findings. With regards to the control variables, it shows that age, gender, and educational-level were associated with stress-related self-efficacy (younger, male, and better-educated participants reported greater stress-related self-efficacy). With regards to social identification as a Hindu, it shows that this had associations with religious practices at home and in temples, and also with perceived standing in the group. However, and of more theoretical significance, it shows that stress-related self-efficacy had positive associations with both perceived standing in the group and social identification as Hindu. Of these associations, the former was stronger (and showed that those who perceived themselves as having greater standing in the community reported greater efficacy in dealing with everyday stressors). Yet, the significance of the latter association constitutes evidence for our key prediction that a stronger social identification as a Hindu would be associated with greater stress-related self-efficacy.

**Table 3:** Factors associated with stress-related self-efficacy (all values are standardized)

Exogenous variables	Endogenous variable	Covariances							
	Stress-related self-efficacy	2.	3.	4.	5	6	7.	8.	9.
1. Social identification as a Hindu	0.14***	0.16***	0.09*	0.20***	−0.04	0.09***	0.15***	−0.02	0.12**
2. Religious practices at home	0.08		0.38***	0.10*	0.10*	0.39***	0.15***	0.01	0.48***
3. Religious practices in temples	0.06			0.09*	0.01	0.00	0.01	−0.02	0.03
4. Perceived standing in the group	0.35***				−0.02	−0.09*	0.15***	−0.04	0.04
5. Age	−0.15***					0.21***	0.02	−0.20***	−0.04
6. Gender	0.10*						−0.03	−0.02	0.62***
7. Caste	0.04							−0.02	0.14***
8. Marital status	−0.00								0.13***
9. Education	0.13**								

*Note*:

* p < .05.

** p < .01

*** p < .001;

Female, low-caste, widowed and illiterate participants were coded zero

### Factors Associated with Well-being

In the second step of the SEM analysis, we investigated the factors associated with our three measures of well-being (self-assessed health, psychological symptoms of ill-health, and physical symptoms of ill-health). Our model specified paths from our key predictor variable—social identification as a Hindu—and the two alternative predictors (religious practices at home and in temples, perceived standing in the group), to the well-being measures *via* stress-related self-efficacy. It also specified direct paths from these variables to the well-being measures. Again, the socio-demographic categories featured as controls (with female, lower caste, widowed, and illiterate participants coded zero). The exogenous variables were allowed to co-vary freely with one another. So too, the well-being measures were allowed to co-vary freely with one another. We used the bootstrapping procedure in AMOS to estimate standard errors and bias-corrected confidence intervals for the direct and indirect estimates in the model (5000 bootstrap resamples and 99% confidence intervals).

**Figure 1:**
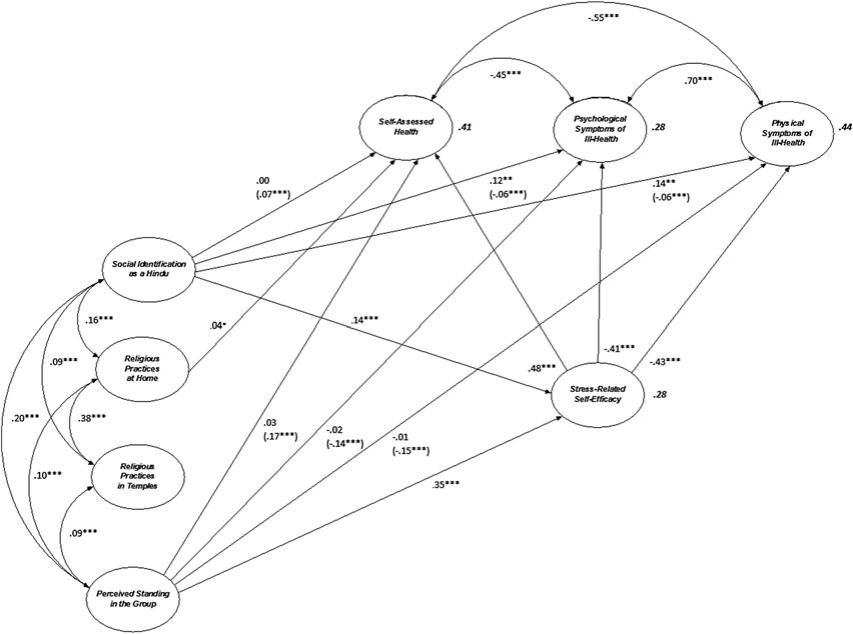
Modelling the relationship between social identification, self-efficacy and well-being: significant paths (direct and indirect) (*p < 0.05 **p < 0.01 ***p < 0.001: indirect effects *via* stress-related self-efficacy are indicated in brackets. All values are standardized)

Although the chi-square statistic was significant (*χ*2 (422) = 1160.20, p < .001), the remaining goodness of fit indices (CFI = 0.94, RMSEA = 0.05 (90% CI: 0.044–0.050), SRMR = 0.04) indicated an acceptable model fit. Figure1 depicts the significant paths in the model (to aid interpretation, the measurement weights, residuals, and structural residuals are not depicted). Tables 4 and 5 report the results relating to the model's direct and indirect paths. The co-variances for the exogenous variables are the same as reported in Table 3, whereas those for the well-being measures feature in Figure1.

**Table 4:** Direct and indirect paths (*via* stress-related self-efficacy): process measures (all values are standardized)

			Endogenous variables			
			Stress-related self-efficacy	Self-assessed health	Psychological symptoms of ill-health	Physical symptoms of ill-health
Social identification as a Hindu	Direct path	β (SE)	0.14*** (0.05)	0.00 (0.06)	0.12** (0.08)	0.14** (0.08)
	Indirect path	β (SE)		0.07***(0.02)	−0.06*** (0.02)	−0.06*** (0.02)
	(LB – UB)	99% CI		(0.033, 0.112)	(−0.097, −0.028)	(−0.103, −0.029)
Religious practices at home	Direct path	β (SE)	0.08 (0.04)	0.04 (0.06)	−0.05 (0.06)	−0.05 (0.06)
	Indirect path	β (SE)		0.04 (0.03)	−0.03 (0.02)	−0.03 (0.03)
	(LB – UB)	99% CI		(−0.033, 0.112)	(−0.099, 0.028)	(−0.103, 0.030)
Religious practices in temples	Direct path	β (SE)	0.06 (0.04)	0.02 (0.01)	0.04 (0.01)	0.02 (0.01)
	Indirect path	β (SE)		0.03 (0.02)	−0.03 (0.02)	−0.03 (0.02)
	(LB – UB)	99% CI		(−0.018, 0.082)	(−0.070, 0.016)	(−0.073, 0.017)
Perceived standing in the group	Direct path	β (SE)	0.35*** (0.04)	0.03 (0.05)	−0.02 (0.07)	−0.01 (0.01)
	Indirect path	β (SE)		0.17*** (0.02)	−0.14*** (0.02)	−0.15*** (0.03)
	(LB – UB)	99% CI		(0.111, 0.231)	(−0.214, −0.086)	(−0.220, −0.090)
Stress-related self-efficacy	Direct effect	β (se)		0.48***(0.06)	−0.41*** (0.07)	−0.43*** (0.07)

*Note*:

*p < .05.

** p < .01

*** p < .001

Confidence Intervals (CI): LB = Lower Bound, UB = Upper Bound

**Table 5:** Direct and Indirect paths (*via* stress-related self-efficacy): socio-demographic categories (all values are standardized)

			Endogenous variables			
Exogenous variables			Stress-related self-efficacy	Self-assessed health	Psychological symptoms of ill-health	Physical symptoms of ill-health
Age	Direct path	β (SE)	−0.15*** (0.00)	−0.18*** (0.00)	0.03(0.00)	0.14*** (0.00)
	Indirect effect	β (SE)		−0.07*** (0.02)	0.06*** (0.02)	0.07*** (0.02)
	(LB – UB)	99% CI		(−0.130, −0.032)	(0.026, 0.113)	(0.028, 0.120)
Gender	Direct path	β (SE)	0.10* (0.07)	0.25*** (0.08)	−0.22*** (0.10)	−0.32*** (0.10)
	Indirect effect	β (SE)		0.05* (0.02)	−0.04* (0.02)	−0.04* (0.02)
	(LB – UB)	99% CI		(−0.005, 0.106)	(−0.099, 0.005)	(−0.100, 0.004)
Caste	Direct path	β (SE)	0.04 (0.09)	−0.04 (0.10)	0.03 (0.13)	−0.02 (0.13)
	Indirect effect	β (SE)		0.02 (0.02)	−0.02 (0.02)	−0.02 (0.02)
	(LB – UB)	99% CI		(−0.028, 0.046)	(−0.063, 0.040)	(−0.066, 0.043)
Marital status	Direct path	β (SE)	−0.00 (0.06)	0.04 (0.07)	−0.00 (0.09)	0.05 (0.09)
	Indirect path	β (SE)		−0.00 (0.02)	0.00 (0.02)	0.00 (0.02)
	(LB – UB)	99% CI		(−0.049, 0.046)	(−0.040, 0.040)	(−0.042, 0.043)
Education	Direct path	β (SE)	0.13** (0.05)	0.01 (0.06)	−0.06 (0.08)	−0.13** (0.08)
	Indirect path	β (SE)		0.06** (0.02)	−0.05** (0.02)	−0.05** (0.02)
	(LB – UB)	99% CI		(0.004, 0.130)	(−0.112, −0.005)	(−0.116, −0.004)

*Note*:

* p < 0.05.

** p < 0.01

*** p < 0.001

Confidence Intervals (CI): LB = Lower Bound, UB = Upper Bound

Consistent with the first step of the SEM analysis reported earlier, Figure1 and Table 4 show direct, positive, and significant paths from perceived standing in the group and social identification as a Hindu to stress-related self-efficacy (with the path from perceived standing in the group being stronger). Religious practices at home and in temples were not associated with stress-related self-efficacy. With regards to well-being, greater stress-related self-efficacy was associated with better outcomes (positive associations with self-assessed health, and negative associations with psychological and physical symptoms of ill-health). None of the direct or indirect paths from religious practices at home and in temples to the well-being measures were significant. Perceived standing in the group was not associated directly with the three well-being measures, but was associated indirectly with these measures *via* stress-related self efficacy.

As explained earlier, we were particularly interested in the association between social identification and well-being *via* stress-related self efficacy. As Table 4 and Figure1 show, we found evidence for indirect associations between social identification as a Hindu and our three well-being measures (self-assessed health, psychological, and physical symptoms of ill-health) *via* greater stress-related self-efficacy. Taken together, these three indirect effects constitute evidence that the level of one's social identification as a Hindu predicts better well-being because this identification is associated with the belief that one can cope with life's stressors.

Our analysis also revealed residual associations between social identification as a Hindu and two of the well-being measures (psychological and physical symptoms of ill-health) after controlling for the pathway through stress-related self-efficacy. These residual associations were more substantial than the indirect effects and showed heightened identification was associated with more psychological and physical symptoms of ill-health. Quite why we found such residual effects indicating an association between greater social identification as a Hindu and *more* psychological and physical symptoms of ill-health is unclear (and is discussed in the succeeding text).

As explained earlier, these analyses included the socio-demographic variables as controls. The direct and indirect associations of these variables are reported in Table 5.^2^ Overall, the model explained 28% of the variance in stress-related self-efficacy, 41% of the variance in self-assessed health and 28% and 44% of the variance in psychological and physical symptoms of ill-health, respectively.

## DISCUSSION

The most theoretically interesting features of these data concern the relationship between participants' social identification as a Hindu—their subjective sense of belonging to a large-scale social group—and their well-being. The correlations in Table 2 pointed to a positive relationship between social identification as a Hindu and one of the measures of well-being (self-assessed health) and to no relationships with the other well-being measures (psychological and physical symptoms of ill-health). However, once we took into account other variables, we found a more complex set of relationships (Table 4). Most importantly, we found that participants' social identification as a Hindu was associated with better well-being through its association with participants' judgements of their own stress-related self-efficacy. That is, as participants' social identification as a Hindu increased they reported greater stress-related self-efficacy and better well-being. Interestingly, this relationship between social identification as a Hindu and better well-being *via* increased stress-related self-efficacy was found for all three measures of well-being (self-assessed health, and lesser psychological and physical symptoms of ill-health). This is consistent evidence that participants' identification with a large-scale group is associated with better well-being *via* their sense of being able to cope in the world.

What makes these associations particularly interesting is that after controlling for the pathway through stress-related self-efficacy, we found residual associations between participants' social identification as a Hindu and two of the three well-being measures (those concerning the psychological and physical symptoms of ill-health). These show that a higher identification was associated with *more* symptoms of ill-health. What lies behind this is unclear. It is possible that a higher identification is associated with practices and routines that take their toll (e.g., higher identifiers may prefer Ayurvedic medicine and be reluctant to take allopathic medicine; Thomas, 1992). However, we also know that people's interpretation of their bodily experiences as ‘symptoms’ and as worthy of concern is bound up with the beliefs and values associated with their social identifications (Levine & Reicher, 1996). Thus, it is conceivable that a higher Hindu identification is associated with a cultural worldview in which psychological and physical experiences are given distinctive meanings and experienced as ‘symptoms’ (and here it may be relevant to note that these associations with a Hindu social identification were only found with the scales designed to capture the somatisation of well-being). Clearly, there is a need for further work, which explores the particular meanings of Hindu identity, the way these affect health-related practices and the way in which bodily experiences are interpreted as ‘symptoms’.

Whatever the reason for these residual associations between participants' social identification as a Hindu and the measures of psychological and physical symptoms of ill-health, the key point to highlight is that we have clear evidence (for all three scales concerning well-being) that a higher social identification as a Hindu had an indirect effect on well-being through its association with the belief that one can cope with everyday stressors (greater stress-related self-efficacy). As we noted in the introduction, there are several mechanisms that might underlie this relationship. For example, social identifications can give life meaning and purpose, provide a sense of continuity, connect one with others, and provide a basis for social support. Future work should address which of these elements is particularly relevant for large-scale social groups and how this depends on their cultural contents.

A further set of questions concern the relative strength of the association between social identification, stress-related self-efficacy, and well-being. As concerns the associations with stress-related self-efficacy, our own analyses show that one's perceived social standing has a stronger link than identification as a Hindu. This is unsurprising: social standing brings innumerable social and material resources, which impact upon the degree to which one may feel efficacious. With regards to the indirect association between social identification and well-being *via* stress-related self-efficacy, it is important to note that the effects are small. However, it is also appropriate to note that the type of coping we explored in our scale is rather general. That is, although our scale is similar to existing measures of stress appraisals used elsewhere (Haslam & Reicher, 2006; Haslam et al., 2009; Reicher & Haslam, 2006a, 2006b), it differs in that our scale emphasizes general as opposed to domain-specific self-efficacy. This makes good sense: we were concerned with a large-scale social identification and the degree to which people felt able to cope with everyday stressors. However, such a lack of specificity is likely to introduce measurement error. Future work on large-scale social identifications could employ a number of scales, each designed to tap more specific domains of life stressors.

In pursuing the relationship between social identification and well-being, future work should ideally have a longitudinal dimension. As with most studies on social identity and well-being, our research was cross-sectional. The limits of such a design are underlined by research showing that one's level of identification with a social group can itself be a function of the degree to which it satisfies particular identity motives (Vignoles, Regalia, Manzi, Golledge, & Scabini, 2006). Future work should also consider the manner in which stress-related self-efficacy impacts on well-being. It may be that those who feel in control of their lives flourish, obtain better resources, and hence experience better well-being (Chiu, Hsu, & Wang, 2006; Podolny & Baron, 1997). It may be they undertake less risky behaviors (Luszczynska, Gutiérrez-Doña, & Schwarzer, 2005; Schwarzer, 2008; Schwarzer & Fuchs, 1996) and thereby experience better well-being. They may also exhibit better functioning of the immune system (Figueredo, 2009; Segerstrom & Miller, 2004).

Although these questions concerning the further specification of the link between a Hindu identification and well-being remain, it is important not to lose sight of what we have shown. Three important conclusions can be drawn from our study. First, large-scale as well as small-scale social identities can be associated with well-being, and this is true not only when the group faces particularly demanding circumstances but also in everyday life. Second, these identifications can have complex and contradictory associations with well-being: identifying with a group can be associated with poorer outcomes as well as with better outcomes. That is, although a social identification can be associated with the sense that one is able to meet the challenges thrown up by everyday life, and thus be indirectly associated with better outcomes, the same social identification may for other reasons be associated with poorer outcomes. Third, on the importance of social identity processes and their relevance to the well-being of ordinary people, we show this is not limited to the urban western world where most work has been conducted. This provides a robust test of a social identity approach to well-being. It would certainly be plausible to suggest that in poorer countries where people are more exposed to harmful material conditions and where health systems are less organized, then social identity processes would be of less relevance to explaining well-being. However, it seems that wherever we go, whether we look at small work-groups or large social categories, at highly stressful circumstances or mundane settings, social identity cannot be discounted as a factor in well-being.

## Footnotes

1 In order to examine the equivalence of this factor structure across the socio-demographic categories recorded in this study, we conducted a Multigroup Analysis in AMOS 17.0 (Arbuckle, 2008). These results are presented in the supplementary materials associated with this paper. We found complete configural and metric invariance, and partial scalar and residual invariance, across the respective socio-demographic categories recorded in the study.

2 The associations between the socio-demographic variables, stress-related self-efficacy, and the well-being outcome measures reported in Table 5 are discussed in the supplementary materials associated with this paper.

## SUPPORTING INFORMATION

Supporting info item

Supporting info item
