# Supplementary figures and images for: Efficacy and well-being in rural north India: The role of social identification with a large-scale community identity
Source: Eur J Soc Psychol. 2014 Aug 25;44(7):787–98. doi: 10.1002/ejsp.2060 (PMC4489324; doi:10.1002/ejsp.2060)

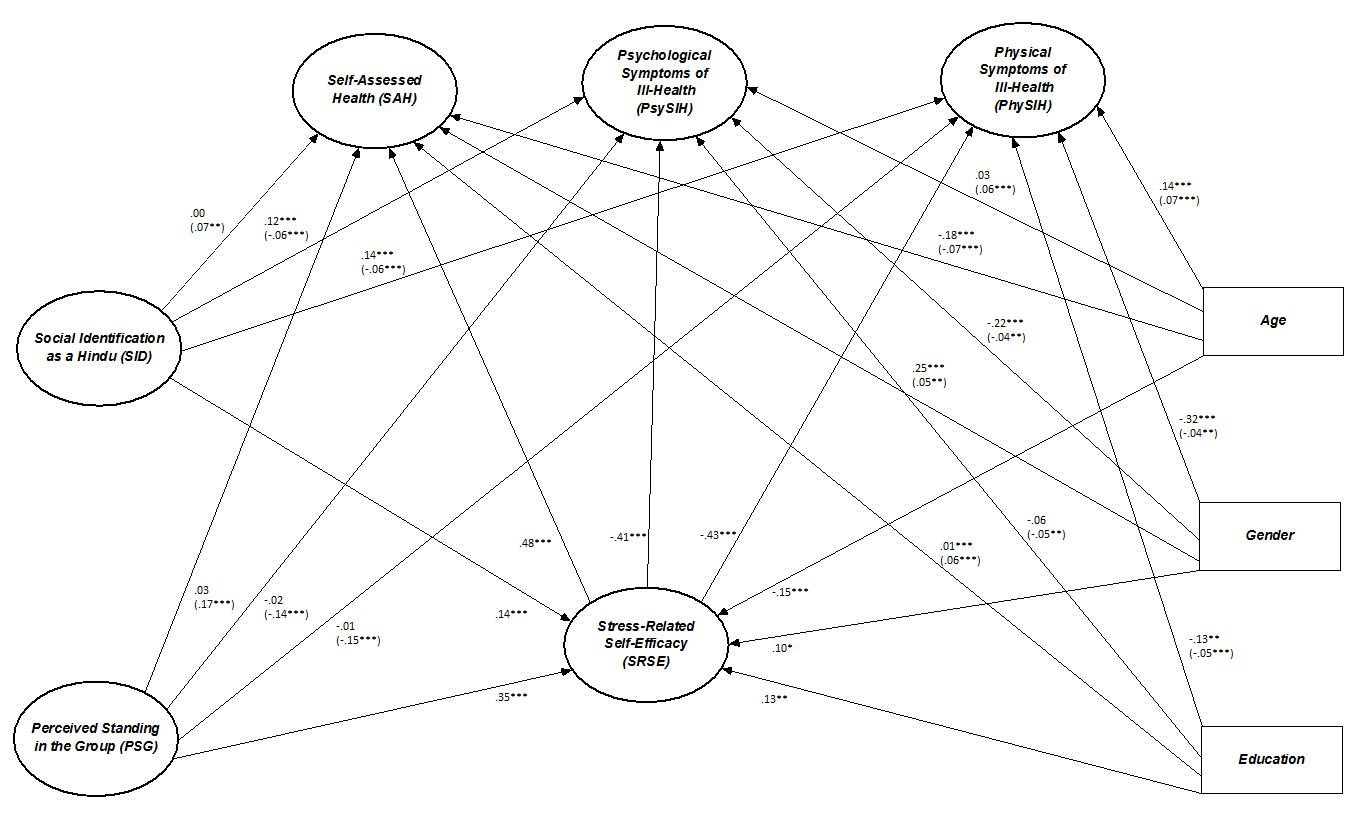

Supplement: Supplementary file 1 — Supporting info item [file ejsp0044-0787-sd1.tif]
