# Supplementary material for: Efficacy and well-being in rural north India: The role of social identification with a large-scale community identity
Source: Eur J Soc Psychol. 2014 Aug 25;44(7):787–98. doi: 10.1002/ejsp.2060 (PMC4489324; doi:10.1002/ejsp.2060)
Supplement: Supplementary file 2 — Supporting info item [file ejsp0044-0787-sd2.doc]

Supplementary Materials

*Multigroup Analyses*

In order to examine if the specified factor structure was equivalent for the different socio-demographic groups recorded in the study (i.e., age, gender, caste, marital status and education) we conducted a series of Multigroup Analyses (MGA) in AMOS 17.0 (Arbuckle, 2008). The MGAs assessed invariance in the measurement model by comparing the levels within each respective socio-demographic variable (e.g., male vs female participants, low-caste vs high-caste participants, etc.). This analytical strategy entailed five separate MGAs.

The first step in each MGA involved establishing a baseline model in which the parameters were estimated freely between the socio-demographic categories. This model is referred to as the configural model and establishes if there is a good fit in the factor structure between groups when no constraints are imposed. Configural invariance (or equivalence) is also a precondition for the comparison of increasingly constrained model parameters between groups. The results from the five MGAs indicated there to be configural invariance across the respective socio-demographic categories (see Table A). Indeed, the measurement weights were statistically significant for every item and socio-demographic category recorded in the study (see Table B). We therefore concluded that there were no striking differences in how participants belonging to the different socio-demographic categories understood and responded to the measures and items administered in the study.

The configural model was in turn used as a baseline for comparisons with nested models in which parameters were increasingly constrained between the respective socio-demographic categories (see Fischer & Fontaine, 2010, Milfont & Fischer, 2010 and Vandenberg & Lance, 2000). We increasingly constrained the measurement weights, measurement intercepts and measurement residuals in the five MGAs.

Cheung and Rensvold (2002) recommend that a CFI difference of less than .01 between each increasingly constrained model with its preceding model (beginning with the unconstrained model) is indicative that the null hypothesis of invariance should accepted. Following these criteria, we found metric equivalence for every socio-demographic variable, scalar equivalence for every socio-demographic variable with the exception of education, and error variance equivalence amongst every socio-demographic variable with the exception of gender and education. Additional item-wise and category-wise examinations indicated that the non-invariance observed in the scalar intercepts for education and error-variances for gender and education was not attributable any one specific category or item but was the result of minor non-invariance across the items within the respective constructs between the respective categories.

Table A reports the goodness-of-fit indices and model comparisons from the five MGAs. Table B reports the measurement weights from the unconstrained (configural) model for every socio-demographic category recorded in the study.

*The Associations between the Socio-Demographic Variables and Well-Being*

Figure A illustrates the SEM with all of the significant predictors, including the socio-demographic variables, of both Stress-Related Self-Efficacy and the three well-being measures. For ease of interpretation, the measurement weights and residuals, and structural co-variances and residuals, are not depicted in the figure.

Our findings concerning the socio-demographic variables recorded in the study show that age, gender and education were significantly associated with our process measure (Stress-Related Self-Efficacy) and our three well-being measures. Younger, male and educated participants exhibited higher levels of Stress-Related Self-Efficacy and (on some of the well-being measures) better well-being. Indirect effects (via Stress-Related Self-Efficacy) on well-being were apparent for Age, Gender and Education. This shows that the association between being younger, male and educated and well-being was (in part) through associations with Stress-Related Self-Efficacy.

One of the more striking features of these indirect effects concerns education. Previous research in India has shown that education can have unique effects on well-being beyond poverty (Rajan, Kennedy & King, 2013) and our data again point to the importance of education. However, our data do not allow us to explore the complex relationship between educational level and other socio-structural factors (e.g., income level).

Finally, it is worth noting that caste was not associated with Stress-Related Self-Efficacy or well-being. However, as other Indian research reports caste differences in health and well-being (e.g., Baru, Acharya, Acharya, Kumar, & Nagaraj 2010; Borooah, 2010; Chen, 1998; Jensen, 2005), it is appropriate to note that our sample did not include many from the lower echelons of the Indian caste system. The degree to which lower caste individuals may gain psychologically from a high Hindu identification requires further investigation (especially as lower caste individuals can experience discrimination and marginalisation).

REFERENCES

Arbuckle, J. L. (2008). Amos (Version 17.0) [Computer Program]. Chicago: SPSS.

Baru, R., Acharya, A., Acharya, S., Kumar, A. K. S., & K, Nagaraj (2010). Inequities in access to health services in India. Caste, class and region. *Economic & Political Weekly, XLV (38)*, 49-58

Borooah, V. K. (2010). Inequality in health outcomes in India: The role of caste and religion. In S. Thorat & K. Newman (Eds.), *Blocked by caste: economic discrimination in modern India* (pp. 179-207). Oxford University Press.

Chen, M., & Dreze, J. (1992). Widows and health in rural north India. *Economic and Political Weekly*, 27, 81-93.

Cheung, G. W., & Rensvold, R. B. (2002). Evaluating goodness-of-fit indexes for testing measurement invariance. *Structural* *Equation Modeling, 9*, 233-255. DOI: DOI:10.1207/S15328007SEM0902_5

Fischer, R., & Fontaine, J. (2010). Methods for investigating structural equivalence. In D. Matsumoto & F. J. van de Vijver (Eds.), *Cross-cultural research methods in psychology* (pp. 179–215). Cambridge, UK: Cambridge University Press.

Jensen, R. T. (2005). Caste, culture and the status and well-being of widows in India. In D. Wise (Ed), *Issues in the Economics of Aging* (pp 357-376). Chicago: University of Chicago Press.

Milfont, T. L., & Fischer, R., (2010). Testing measurement invariance across groups: Applications in cross-cultural research. *International Journal of Psychological Research, 3,* 111-121. Retrieved from <http://mvint.usbmed.edu.co:8002/ojs/index.php/web/article/view/465/449>

Rajan, K., Kennedy, J. & King, L. (2013). Is wealthier always healthier in poor countries? The health implications of income, inequality, poverty, and literacy in India. *Social Science & Medicine, 88,* 98-107 DOI: 10.1016/j.socscimed.2013.04.004

Vandenberg, R.J., & Lance, C.E. (2000). A review and synthesis of the measurement invariance literature: Suggestions, practices and recommendations for organizational research. *Organizational Research Methods, 3,* 4-70. DOI: 10.1177/109442810031002

Table A: Results from Multigroup Analysis (MGA) Factor Structure Specified in Confirmatory Factor Analysis (CFA)

|  | *df* | *χ^2^* | *χ2/df* | *CFI* | *RMSEA* | *SRMR* | *∆χ^2^* | *∆CFI* | *∆RMSEA* | *∆SRMR* |
| --- | --- | --- | --- | --- | --- | --- | --- | --- | --- | --- |
| *Age* |  |  |  |  |  |  |  |  |  |  |
| *Unconstrained (Configural)* | 644 | 1269.19 | 1.97 | .95 | .04 | .03 |  |  |  |  |
| *Measurement Weights* | 664 | 1294.80 | 1.95 | .95 | .04 | .04 | 25.61ns | .00 | .00 | .01 |
| *Measurement Intercepts* | 692 | 1363.19 | 1.97 | .95 | .04 | .04 | 68.39*** | .00 | .00 | .00 |
| *Measurement Residuals* | 756 | 1466.68 | 1.94 | .94 | .03 | .04 | 103.49*** | .01 | .01 | .00 |
|  |  |  |  |  |  |  |  |  |  |  |
| *Gender* |  |  |  |  |  |  |  |  |  |  |
| *Unconstrained (Configural)* | 644 | 1348.23 | 2.09 | .94 | .03 | .04 |  |  |  |  |
| *Measurement Weights* | 664 | 1438.93 | 2.17 | .93 | .04 | .04 | 90.70*** | .01 | .01 | .00 |
| *Measurement Intercepts* | 692 | 1831.02 | 2.65 | .92 | .04 | .05 | 392.01*** | .01 | .00 | .01 |
| *Measurement Residuals* | 756 | 2216.47 | 2.93 | .88 | .05 | .05 | 385.45*** | .04 | .01 | .00 |
|  |  |  |  |  |  |  |  |  |  |  |
| *Caste* |  |  |  |  |  |  |  |  |  |  |
| *Unconstrained (Configural)* | 644 | 1315.32 | 2.04 | .95 | .04 | .03 |  |  |  |  |
| *Measurement Weights* | 664 | 1348.89 | 2.03 | .94 | .04 | .03 | 33.57*** | .01 | .00 | .00 |
| *Measurement Intercepts* | 692 | 1403.64 | 2.03 | .94 | .04 | .03 | 54.75*** | .00 | .00 | .00 |
| *Measurement Residuals* | 756 | 1539.51 | 2.04 | .94 | .04 | .04 | 135.87*** | .00 | .00 | .01 |
|  |  |  |  |  |  |  |  |  |  |  |
| *Marital Status* |  |  |  |  |  |  |  |  |  |  |
| *Unconstrained (Configural)* | 644 | 1337.49 | 2.08 | .94 | .04 | .04 |  |  |  |  |
| *Measurement Weights* | 664 | 1362.91 | 2.05 | .94 | .04 | .04 | 25.42ns | .00 | .00 | .00 |
| *Measurement Intercepts* | 692 | 1408.93 | 2.04 | .94 | .04 | .04 | 46.02* | .00 | .00 | .00 |
| *Measurement Residuals* | 756 | 1487.61 | 1.97 | .94 | .04 | .04 | 78.68** | .00 | .00 | .00 |
|  |  |  |  |  |  |  |  |  |  |  |
| *Education* |  |  |  |  |  |  |  |  |  |  |
| *Unconstrained (Configural)* | 644 | 1702.87 | 1.76 | .94 | .03 | .05 |  |  |  |  |
| *Measurement Weights* | 664 | 1832.37 | 1.82 | .93 | .03 | .05 | 129.50*** | .01 | .00 | .00 |
| *Measurement Intercepts* | 692 | 2321.05 | 2.19 | .90 | .04 | .05 | 488.69*** | .03 | .01 | .00 |
| *Measurement Residuals* | 756 | 2918.59 | 2.45 | .86 | .04 | .06 | 597.54*** | .04 | .00 | .01 |
|  |  |  |  |  |  |  |  |  |  |  |

* p < .05. ** p < .01 *** p < .001

Table B: Standardised Measurement Weights of the Unconstrained Model in the MGA

|  |  |  | *Social Identification*  *as a Hindu* | | |  | *Religious Practices*  *at Home* | | |  | *Religious Practices*  *in Temples* | | |  | *Perceived Standing*  *in the Group* | | | | |  |
| --- | --- | --- | --- | --- | --- | --- | --- | --- | --- | --- | --- | --- | --- | --- | --- | --- | --- | --- | --- | --- |
|  |  |  | Items | | |  | Items | | |  | Items | | |  | Items | | | | |  |
|  |  |  | *1* | *2* | *3* |  | *1* | *2* | *3* |  | *1* | *2* | *3* |  | *1* | *2* | *3* | *4* | *5* |  |
|  | *Age*  *Below Median*  *Above Median* |  | .75***  .83*** | .94***  .92*** | .82***  .94*** |  | .67***  .62*** | .57***  .62*** | .63***  .62*** |  | .92***  .95*** | .98***  .91*** | .63***  .68*** |  | .90***  .86*** | .89***  .89*** | .88***  .88*** | .85***  .82*** | .85***  .83*** |  |
|  |  |  |  |  |  |  |  |  |  |  |  |  |  |  |  |  |  |  |  |  |
|  | *Gender*  *Male*  *Female* |  | .80***  .79*** | .92***  .92*** | .82***  .85*** |  | .75***  .59*** | .57***  .63*** | .76***  .50*** |  | .94***  .93*** | .88***  1.00*** | .74***  .59*** |  | .88***  .88*** | .89***  .89*** | .86***  .89*** | .82***  .84*** | .84***  .84*** |  |
|  |  |  |  |  |  |  |  |  |  |  |  |  |  |  |  |  |  |  |  |  |
|  | *Caste*  *General*  *OBC* |  | .78***  .84*** | .91***  1.00*** | .82***  .87*** |  | .60***  .91*** | .61***  .57*** | .60***  .39** |  | .94***  1.00*** | .94***  .90*** | .68***  .49*** |  | .86***  .94*** | .89***  .93*** | .86***  .97*** | .83***  .77*** | .84***  .80*** |  |
|  |  |  |  |  |  |  |  |  |  |  |  |  |  |  |  |  |  |  |  |  |
|  | *Marital-Status*  *Married*  *Widowed* |  | .79***  .82*** | .83***  .91*** | .88***  .84*** |  | .64***  .59*** | .58***  .72*** | .66***  .52** |  | .94***  .94*** | .93***  .94*** | .66***  .67*** |  | .88***  .88*** | .90***  .86*** | .87***  .89*** | .83***  .82*** | .83***  .87*** |  |
|  |  |  |  |  |  |  |  |  |  |  |  |  |  |  |  |  |  |  |  |  |
|  | *Education*  *University*  *Primary-Intermediate*  *Illiterate* |  | .77***  .83***  .76*** | .97***  .91***  .94*** | .80***  .84***  .83*** |  | .63***  .68***  .67*** | .64***  .56***  .57*** | .90***  .71***  .39** |  | .96***  .90***  .96*** | .92***  .92***  .98*** | .82***  .73***  .53*** |  | .82***  .88***  .89*** | .88***  .90***  .89*** | .83***  .86***  .90*** | .83***  .81***  .86*** | .84***  .83***  .85*** |  |
|  |  |  |  |  |  |  |  |  |  |  |  |  |  |  |  |  |  |  |  |  |

* p < .05. ** p < .01 *** p < .001

|  |  |  | *Stress-Related*  *Self-Efficacy* | | | | |  | *Self-Assessed*  *Health* | | |  | *Psychological*  *Symptoms*  *of Ill-Health* | | |  | *Physical*  *Symptoms*  *of Ill-Health* | | |  |
| --- | --- | --- | --- | --- | --- | --- | --- | --- | --- | --- | --- | --- | --- | --- | --- | --- | --- | --- | --- | --- |
|  |  |  | Items | | | | |  | Items | | |  | Items | | |  | Items | | |  |
|  |  |  | *1* | *2* | *3* | *4* | *5* |  | *1* | *2* | *3* |  | *1* | *2* | *3* |  | *1* | *2* | *3* |  |
|  | *Age*  *Below Median*  *Above Median* |  | .60***  .71*** | .78***  .81*** | .86***  .88*** | .79***  .77*** | .73***  .82*** |  | .74***  .69*** | .78***  .71*** | .73***  .74*** |  | .82***  .77*** | .89***  .84*** | .74***  .73*** |  | .70***  .67*** | .47***  .46*** | .64***  .62*** |  |
|  |  |  |  |  |  |  |  |  |  |  |  |  |  |  |  |  |  |  |  |  |
|  | *Gender*  *Male*  *Female* |  | .70***  .64*** | .82***  .79*** | .82***  .90*** | .69***  .82*** | .80***  .78*** |  | .79***  .64*** | .76***  .70*** | .74***  .70*** |  | .79***  .76*** | .80***  .88*** | .67***  .74*** |  | .65***  .65*** | .48***  .45*** | .51***  .62*** |  |
|  |  |  |  |  |  |  |  |  |  |  |  |  |  |  |  |  |  |  |  |  |
|  | *Caste*  *General*  *OBC* |  | .66***  .75*** | .80***  .82*** | .87***  .82*** | .77***  .73*** | .78***  .87*** |  | .71***  .85*** | .74***  .82*** | .75***  .66*** |  | .79***  .85*** | .86***  .91*** | .73**  .71*** |  | .62***  .73*** | .46***  .46*** | .62***  .65*** |  |
|  |  |  |  |  |  |  |  |  |  |  |  |  |  |  |  |  |  |  |  |  |
|  | *Marital-Status*  *Married*  *Widowed* |  | .66***  .71*** | .82***  .78*** | .88***  .84*** | .78***  .74*** | .81***  .72*** |  | .72***  .72*** | .75***  .71*** | .75***  .73*** |  | .80***  .74*** | .87***  .87*** | .71**  .82*** |  | .68***  .66*** | .50***  .32*** | .62***  .68*** |  |
|  |  |  |  |  |  |  |  |  |  |  |  |  |  |  |  |  |  |  |  |  |
|  | *Education*  *University*  *Primary-Intermediate*  *Illiterate* |  | .54***  .67***  .65*** | .70***  .80***  .81*** | .88***  .83***  .90*** | .66***  .69***  .82*** | .53***  .77***  .83*** |  | 1.00***  .68***  .67*** | .68***  .74***  .77*** | .63***  .71***  .74*** |  | .76***  .81***  .73*** | .81***  .85***  .88*** | .52***  .70***  .75*** |  | .44***  .65***  .67*** | .52***  .48***  .43*** | .68***  .54***  .60*** |  |
|  |  |  |  |  |  |  |  |  |  |  |  |  |  |  |  |  |  |  |  |  |

Table B Continued: Standardised Measurement Weights of the Unconstrained Model in the MGA

* p < .05. ** p < .01 *** p < .001

Figure A. Modelling the Associations between the Socio-Demographic Variables and Well-Being: Significant paths (direct and indirect)

(* p < .05. ** p < .01 *** p < .001. Indirect effects *via* Stress-Related Self-Efficacy are indicated in brackets. All values are standardised).
